# Improving LIME Stability via Density-Awareness: Evaluation and Comparison of AKDE-LIME

**DOI:** 10.1080/08839514.2026.2640686

**Published:** 2026-03-07

**Authors:** Grigorios Tzionis, Georgia Kougka, Ilias Gialampoukidis, Stefanos Vrochidis, Ioannis Kompatsiaris, Maro Vlachopoulou

**Affiliations:** a Information Technologies Institute (ITI), Centre for Research and Technology Hellas (CERTH), Thessaloniki, Greece; b Department of Applied Informatics, University of Macedonia, Thessaloniki, Greece

## Abstract

This paper addresses the critical instability of Local Interpretable Model-agnostic Explanations (LIME). We introduce Adaptive Kernel Density Estimation LIME (AKDE-LIME), a novel approach that enhances local explanation stability by incorporating a density-aware weighting scheme. Unlike LIME’s standard proximity kernel, AKDE-LIME combines distance weighting with a Kernel Density Estimate (KDE) of the local sample distribution, assigning more representative weights to generated perturbations. We conduct a comprehensive evaluation of AKDE-LIME against LIME, TreeSHAP, and Anchor on five diverse tree-based models using a real-world dataset. Assessing performance on Stability and Robustness metrics across a matrix of noise levels (5% to 20%), our results consistently demonstrate that AKDE-LIME produces significantly more stable and robust explanations than standard LIME under all conditions. The performance of our method is often comparable to or better than state-of-the-art explainers like TreeSHAP. We conclude that AKDE-LIME is a promising and reliable alternative for generating trustworthy local explanations, addressing a key weakness of the original LIME algorithm.

## Introduction

The proliferation of complex machine learning models, particularly deep neural networks (LeCun, Bengio, and Hinton 2015) and large ensemble methods such as gradient boosting (Chen and Guestrin 2016), has led to significant advancements in predictive accuracy across numerous domains. However, the increasing complexity of these models often comes at the cost of interpretability, creating “black box” systems whose decision-making processes are opaque to human users. This lack of transparency poses a significant challenge in high-stakes fields such as finance, healthcare, and autonomous systems, where trust, accountability, and the ability to debug model behavior are paramount (Doshi-Velez and Kim 2017). Consequently, the field of Explainable AI (XAI) has emerged to develop methods that render these models more understandable to humans (Adadi and Berrada 2018).

Within XAI, a prominent category of techniques provides local, model-agnostic explanations, which aim to explain individual predictions without requiring access to the model’s internal structure. One of the most influential methods in this area is LIME (Rudin 2019; Tulio Ribeiro, Singh, and Guestrin 2016). LIME operates by learning a simple, interpretable model (e.g., a linear model) on a neighborhood of perturbed samples generated around the instance to be explained. The weights of this local surrogate model are then used as feature importance scores. While powerful and widely adopted, a critical limitation of LIME is the potential instability of its explanations; different samplings of the local neighborhood can lead to substantially different explanations for the same prediction, undermining user trust (Alvarez-Melis and Jaakkola 2018).

To illustrate this instability conceptually, consider a simple two-dimensional classification task with a non-linear decision boundary. If we wish to explain the prediction for an instance x located near this boundary, LIME generates random perturbations around x. Due to the random nature of this sampling and the curvature of the boundary, one set of perturbations might fall predominantly on one side of the decision surface, leading the local linear surrogate model to assign importance primarily to one feature. However, a slightly different random sample might fall mostly on the other side, potentially resulting in a different linear approximation that emphasizes the other feature. Furthermore, standard LIME’s proximity weighting treats all points at a similar distance equally, even if the underlying data distribution is much denser in certain directions around x. This sensitivity to sampling and lack of awareness of local data density can lead to inconsistent explanations for the same instance x, making it difficult for users to rely on the generated feature importances. Addressing this requires a method that produces explanations more robust to sampling variations, potentially by incorporating information about the local data structure.

Motivated by this challenge, in this paper, we propose AKDE-LIME, a novel approach designed to improve the stability and robustness of local explanations. Our method enhances the standard LIME framework by introducing a more sophisticated, density-aware weighting scheme. Instead of weighting perturbed samples based solely on their proximity to the original instance, AKDE-LIME combines this proximity measure with a KDE derived from the distribution of distances in the local neighborhood. The core hypothesis is that by giving higher weight to perturbations that lie in denser, more representative regions of the local data manifold, the resulting surrogate model will be less sensitive to sampling artifacts and thus produce more stable feature attributions.

We conduct a comprehensive and systematic evaluation to validate the performance of AKDE-LIME. We compare its stability and robustness against three state-of-the-art methods: standard LIME (Tulio Ribeiro, Singh, and Guestrin 2016), TreeSHAP (Lundberg and Lee 2017), and Anchor (Tulio Ribeiro, Singh, and Guestrin 2018). These methods were selected to provide a comprehensive benchmark: LIME serves as the direct baseline for our enhancement, TreeSHAP represents a theoretically grounded, state-of-the-art feature attribution technique for tree models, and Anchor offers a contrasting rule-based explanation paradigm known for its stability. This comparison is performed across five distinct, commonly used tree-based models: Decision Tree, Random Forest, Extra Trees, LightGBM, and HistGradientBoostingClassifier, using the real-world Steel Industry dataset (V E, Shin, and Cho 2021). The evaluation is conducted across a matrix of experiments, systematically varying the level of input noise (5%, 10%, 15%, 20%) and the number of instances sampled for evaluation (10 to 50), providing a thorough assessment of each explainer’s performance under different conditions.

Our contributions are threefold: 1) We propose AKDE-LIME, a novel, density-aware local explanation method. 2) We present a rigorous experimental framework for comparing the stability and robustness of local explainers. 3) We demonstrate through extensive experiments that AKDE-LIME consistently yields more stable and robust explanations than standard LIME and exhibits a level of stability comparable to or exceeding other leading methods.

The remainder of this paper is structured as follows: “Related Work” reviews related work in local explanations. “Methodology” describes the overall methodology, including the baseline explainers and evaluation metrics. “Proposed Method: AKDE-LIME” details our proposed AKDE-LIME method. “Experimental Setup” outlines the experimental setup. Finally, “Limitations” discusses the limitations of this study, and “Conclusions” concludes with a summary of our findings and directions for future work.

## Related work

The pursuit of explainable AI has given rise to a diverse array of methods, each with distinct approaches to demystifying black-box models. Our work is situated within the context of local, model-agnostic explainers and the critical evaluation of their properties. This section reviews the foundational techniques relevant to our study, including local surrogate models, Shapley value-based methods, and the evaluation criteria used to assess their quality.

### Local surrogate model-agnostic explainers

A significant portion of XAI research focuses on local, model-agnostic methods, which explain individual predictions of a model without needing access to its internal architecture. These methods treat the primary model as a black box, making them highly versatile.

LIME, proposed by Ribeiro, Singh, and Guestrin (Tulio Ribeiro, Singh, and Guestrin 2016), is a pioneering technique in this category. The core principle of LIME is to approximate the behavior of any complex model in the local vicinity of a specific prediction with a simpler, interpretable surrogate model, such as a linear model or a decision tree. To achieve this, LIME generates a neighborhood of perturbed data points around the instance to be explained. These points are then weighted based on their proximity (typically using an exponential kernel) to the original instance. The learned surrogate model provides a locally faithful explanation in the form of feature importance scores. While widely influential due to its intuitiveness and applicability, a key drawback of LIME is its potential for instability. Studies have shown that the explanations can vary significantly based on the random sampling of the neighborhood and the choice of the kernel width, which can undermine user trust (Alvarez-Melis and Jaakkola 2018; Zhou, Hooker, and Wang 2021). Our work directly addresses this limitation by proposing a more robust weighting mechanism.

Several extensions tackle this instability head-on. S-LIME maximizes an explicit stability objective during sampling (Zhou, Hooker, and Wang 2021), while MAPLE (Plumb, Molitor, and Talwalkar 2018) uses a random-forest density estimator to weight perturbations, yielding smoother local models. Other variants, such as Kernelised LIME (KL-LIME) (Nematzadeh et al. 2023), adopt Bayesian or adversarial sampling schemes to tighten neighborhood fidelity (Nematzadeh et al. 2023). Our proposed AKDE-LIME fits into this family: it augments LIME’s proximity kernel with an adaptive KDE term, emphasizing perturbations that fall in dense, representative regions of the local manifold.

Anchor provides an alternative form of local explanation based on high-precision rules (Tulio Ribeiro, Singh, and Guestrin 2018). Developed by the same authors as LIME, Anchor aims to find a minimal set of IF-THEN rules (the “anchor”) that “locks” the model’s prediction locally. This means that for any perturbed instance where the anchor rule holds, the model’s prediction is guaranteed to remain the same with a high probability (the “precision”). Unlike LIME’s feature attributions, Anchor provides clear, understandable conditions for a prediction, but it does not inherently rank the importance of features outside of the generated rule.

### Shapley value-based explanations

An alternative and theoretically grounded approach to feature attribution is based on Shapley values, a concept originating from cooperative game theory. SHAP, introduced by Lundberg and Lee (Lundberg and Lee 2017), provides a unified framework for interpreting model predictions by assigning each feature an importance value representing its marginal contribution to the final output. SHAP values have desirable theoretical properties, including local accuracy and consistency, making them a robust method for feature attribution.

While the model-agnostic KernelSHAP can be applied to any model, it is expensive; for tree ensembles we therefore use TreeSHAP, an exact, polynomial-time algorithm (Lundberg et al. 2020). Recent work continues to push efficiency, introducing GPU-accelerated implementations (Mitchell, Frank, and Holmes 2022) and distillation-based proxies such as FastSHAP (Jethani et al. 2021). These methods serve as strong baselines in our experiments. For the tree-based models used in our experiments, we utilize TreeSHAP, a highly efficient and model-specific implementation. TreeSHAP calculates exact Shapley values by leveraging the internal tree structures of these models, providing fast and consistent explanations without the need for perturbation sampling (Lundberg et al. 2020). In our comparative study, TreeSHAP serves as a powerful baseline representing a state-of-the-art feature attribution method.

### Evaluating the quality of explanations

Across the literature, explanations are judged along several axes – fidelity, stability, robustness, and user utility. Doshi-Velez and Kim set out early desiderata for a rigorous science of interpretability (Doshi-Velez and Kim 2017), while Hooker et al. introduce stress tests for saliency maps (Hooker et al. 2019). We adopt the quantitative metrics proposed in those works wherever possible and extend them with the KDE-weighted stability score introduced by Zhou et al. (Zhou, Hooker, and Wang 2021).

#### Metrics for this study

In this paper, we focus on empirically measuring the following two properties:

- **Stability** (also referred to as consistency or self-similarity) measures how much an explanation changes due to the method’s own internal randomness (e.g., random sampling), Equation 1. A stable explainer should produce similar outputs for the same input instance across multiple runs (Zhou, Hooker, and Wang 2021).
  (1) $$S=\frac{1}{N}\sum_{i=1}^{N}\frac{|r_{i}-r_{i{\;}^{\prime }}|}{K}$$
- **Robustness** measures how much an explanation changes in response to small, meaningful perturbations of the input instance, Equation 2. A robust explanation should not change erratically when the input is slightly modified, reflecting a smooth and reliable interpretation of the model’s local behavior (Alvarez-Melis and Jaakkola 2018).
  (2) $$R=\frac{1}{N}\sum_{i=1}^{N}\frac{|w_{i}-{w{\;}^{\prime }}_{i}|}{|w_{i}|}$$

We specifically selected these metrics not only for their importance in establishing user trust but also for their universal applicability across the different types of explainers under review, from feature attribution methods to rule-based systems. By evaluating LIME, AKDE-LIME, TreeSHAP, and Anchor against these crucial metrics, our study contributes to the systematic assessment of XAI techniques, providing empirical evidence to guide practitioners in selecting the most reliable method for their needs.

## Methodology

To empirically evaluate the performance of our proposed AKDE-LIME method, we designed a systematic experimental framework to compare its stability and robustness against other leading local explanation techniques. This section details the components of our methodology, including the dataset and preprocessing steps, the machine learning models used, the formulation of the baseline and proposed methods, and the specific metrics used for evaluation.

### Dataset and preprocessing

The experiments were conducted on the “Steel Industry Energy Consumption” dataset (V E, Shin, and Cho 2021), a real-world time-series dataset containing energy usage metrics.

**Features** We feed the classifier with seven continuous predictors:

• Usage_kWh

• Lagging_Current_Reactive_Power_kVarh

• Leading_Current_Reactive_Power_kVarh

• CO ${\;}_{2}$ (tCO ${\;}_{2}$)

• Lagging_Current_Power_Factor

• Leading_Current_Power_Factor

• NSM – seconds elapsed since midnight

All date-related columns and weekday/weekend flags are discarded.

**Response variable** Load_Type is a categorical label with three states:

• Light Load

• Medium Load

• Maximum Load

These classes are label-encoded as integers before model training.

### Black-Box models

To ensure a comprehensive evaluation, we tested the explanation methods on a diverse set of five commonly used, high-performing tree-based ensemble models. The models selected were:

1. Decision Tree (DecisionTreeClassifier)
2. Random Forest (RandomForestClassifier)
3. Extra Trees (ExtraTreesClassifier)
4. LightGBM (LGBMClassifier)
5. CatBoost (CatBoostClassifier)

These models represent a range of algorithmic approaches, from single trees to bagging and various gradient boosting frameworks, providing a robust testbed for evaluating the model-agnostic capabilities of the XAI methods. Each model was trained on the preprocessed training data using its default or standard hyperparameters.

### Explanation methods

#### Baseline explainers

- LIME (Tulio Ribeiro, Singh, and Guestrin 2016) explains a prediction by approximating the black-box model f with a simpler, interpretable surrogate model g (e.g., a linear model) in the local neighborhood of the instance x to be explained, Equation 3. The objective is to minimize the following function:
  (3) $$\underset{g\in G}{\mathrm{minimize}}\left(L(f,g,\pi_{x})+\Omega (g)\right)$$
  where L is a fidelity function measuring how well g approximates f in the neighborhood defined by the proximity kernel $\pi_{x}$, and Ω(g) is a penalty term for model complexity. The weight kernel $\pi_{x}$ typically takes the form of an exponential kernel based on a distance function D, Equation 4:
  (4) $$\pi_{x}(z)=\exp\left(-\frac{D{\left(x,z\right)}^{2}}{\sigma^{2}}\right)$$
- SHAP (Lundberg and Lee 2017) is a method based on cooperative game theory that assigns each feature an importance value, or Shapley value, representing its contribution to a prediction. For tree-based models, the highly efficient TreeSHAP algorithm (Lundberg and Lee 2017) calculates these values exactly, Equation 5. An explanation is an additive feature attribution model where the prediction f(x) is approximated by a linear function of binary variables $z{\;}^{\prime }\in \{0,1{\}}^{M}$:
  (5) $$f(x)\approx g(z{\;}^{\prime })=\phi_{0}+\sum_{i=1}^{M}\phi_{i}z{{\;}^{\prime }}_{i}$$
  where M is the number of input features, $\phi_{0}$ is the base value (average model output over the training data), and $\phi_{i}$ is the Shapley value for feature i.
- Anchor method (Tulio Ribeiro, Singh, and Guestrin 2018) produces high-precision, rule-based explanations. An anchor is a set of IF-THEN rules (predicates on features) that sufficiently “anchors” the prediction locally. The algorithm aims to find a rule A such that for other instances z where the rule holds (A(z)=1), the model’s prediction is highly likely to be the same as for the original instance x, Equation 6. The objective is to maximize precision subject to a minimum coverage constraint τ:
  (6) $$\underset{As.t.A(x)=1}{\mathrm{maximize}}\left(E_{z∼D(z|A(z))}[I(f(x)=f(z))]\right)\;s.t.\;E_{z∼D(z)}[A(z)]\geq \tau$$

where D is the distribution of perturbations around x.

## Proposed Method: AKDE-LIME

**Figure 1.**
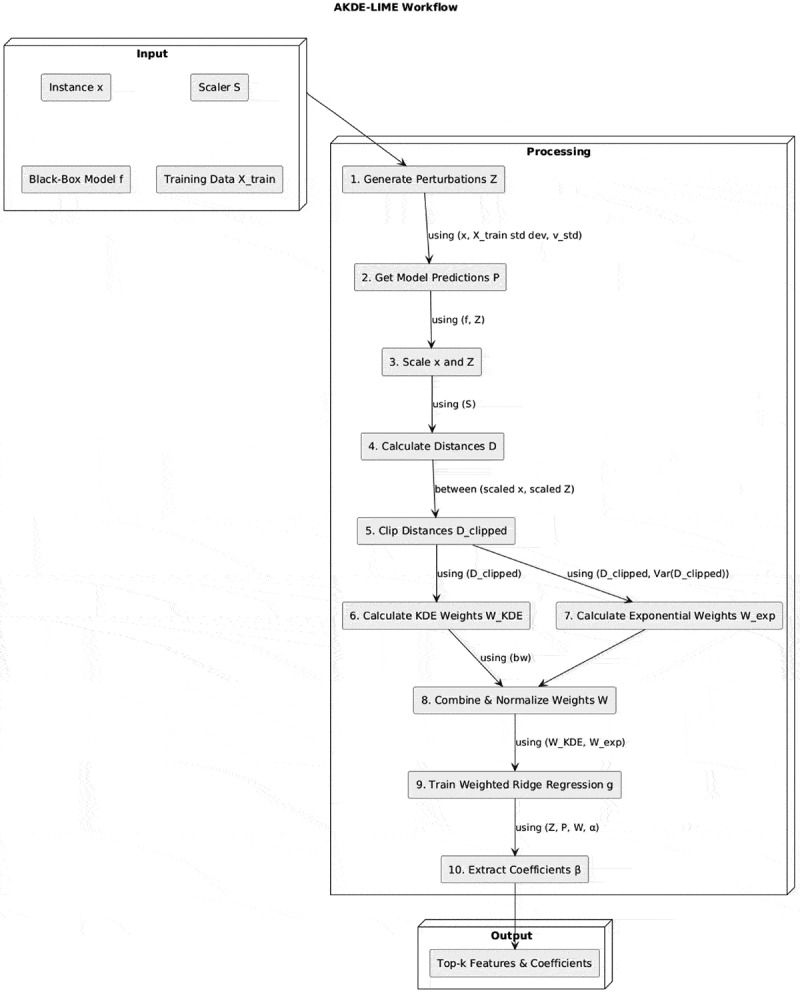
Architectural overview of the proposed AKDE-LIME method.

Our proposed method, AKDE-LIME, enhances the standard LIME framework by integrating an adaptive weighting scheme based on KDE. Figure 1 provides a schematic overview of the AKDE-LIME process, illustrating the flow from input instance to the final explanation. Perturbed samples are generated, weighted based on both scaled KDE, and used to train a local Ridge surrogate model whose coefficients provide the feature importance scores.

The full algorithm is detailed in Algorithm 1. The core of the method is the objective function for training the weighted Ridge Regression surrogate model. The coefficients b of this surrogate model serve as the feature importances, as shown in Equation 7:

(7) $$\underset{\beta_{0},\beta }{\mathrm{minimize}}\left(\sum_{j=1}^{N}w_{j}{\left(f{\left(z_{j}\right)}_{c}-(\beta_{0}+z_{j}\cdot \beta )\right)}^{2}+\alpha ||\beta |{|}_{2}^{2}\right)$$

where the adaptive weight $w_{j}$ for each perturbed sample $z_{j}$ is a product of a standard exponential kernel and a KDE-based density term (Equation 8):

(8) $$w_{j}\propto \exp\left(-\frac{d_{j,c\mathrm{lipped}}^{2}}{\mathrm{Var}(D_{\mathrm{clipped}})}\right)\times \mathrm{KDE}(d_{j,\mathrm{clipped}})$$

Standard LIME uses a fixed exponential kernel, $w_{j}^{LIME}=\exp(-d_{j}^{2}/\sigma^{2})$. When the local data manifold is heterogeneous (e.g., dense clusters next to sparse regions), this kernel can over-weight isolated samples that happen to lie close to the query instance x, potentially producing erratic coefficients. AKDE-LIME rescales that kernel with a density term $\hat{p}(d_{j}|\mathrm{bw})$ obtained via 1-D KDE on the distance vector D. This approach rewards perturbations located in *dense* areas around x and penalizes outliers. The clipping at the 99th percentile prevents very large distances from dominating the bandwidth estimation, and using the variance $\mathrm{Var}(D_{\mathrm{clipped}})$ in the exponential kernel adaptively stabilizes the scale.

The hyperparameter tuple $\left\langle N,\mathrm{bw},\alpha ,v_{\mathrm{std}}\rangle =\langle 1000,0.2,1,0.01\right\rangle$ was chosen via a coarse grid search on a held-out validation fold. Our empirical results suggest that performance is relatively insensitive to bw∈[0.1,0.5] and $v_{\mathrm{std}}\in [0.005,0.05]$. The Ridge penalty α was observed to primarily control numerical stability rather than fidelity.

AKDE-LIME introduces O(N) overhead for the KDE evaluation. The weighted Ridge surrogate is solved in closed form with a cost of $O(Nd^{2})$. For $N=10^{3}$ and d<50, the entire explanation is computed in under $10^{-2}$ seconds on a standard laptop (see Appendix C for details).

AKDE-LIME is particularly beneficial near class boundaries or in highly imbalanced datasets where standard LIME coefficients are known to fluctuate markedly across runs. In uniformly-dense regions, the KDE term becomes flat, and AKDE-LIME gracefully reduces to vanilla LIME, incurring negligible extra cost.

**Table:** 

**Algorithm 1** AKDE-LIME explanation.
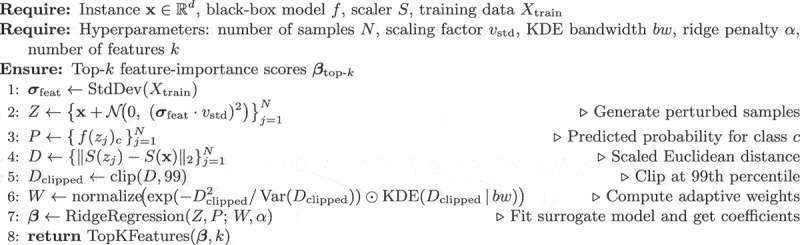

### Evaluation protocol and implemented metrics

To empirically assess the quality of the explanations, we define both an evaluation protocol outlining the experimental procedure and specific evaluation metrics used for quantitative measurement.

The evaluation protocol specifies the overall methodology for testing stability and robustness consistently across explainers. For each ML model, we performed a grid of experiments, varying the input noise level ({0.05,0.10,0.15,0.20}) and the number of test instances evaluated ({10,20,30,40,50}).

The evaluation process for a single instance is as follows:

1. A base explanation is generated for the original, unperturbed instance.
2. Gaussian noise, scaled by the specified noise level, is added to the instance five times to create five unique perturbed versions.
3. An explanation is generated for each of the five perturbed versions.
4. The dissimilarity between the base explanation and each of the five noisy explanations is calculated.
5. The average of these five dissimilarity scores constitutes the final score for that instance. This score is then averaged across all tested instances to yield the final metric.

The dissimilarity in step 4 is calculated using specific evaluation metrics, chosen for their applicability and interpretability. While formal metrics evaluating stability and robustness exist, such as rank correlation for stability (Zhou, Hooker, and Wang 2021) (Equation 1) and relative change in importance for robustness (Alvarez-Melis and Jaakkola 2018) (Equation 2), they present practical challenges in a comparative study involving diverse explanation types. Specifically, rank-based metrics like Equation 1 are ill-suited for rule-based explainers like Anchor, which do not produce ranked feature lists. Furthermore, relative change metrics like Equation 2 become undefined or numerically unstable when a feature’s base importance ($w_{i}$) is zero or near-zero.

While many such criteria exist, the work of Alvarez-Melis and Jaakkola (Alvarez-Melis and Jaakkola 2018) highlighted that *robustness* is a particularly critical, yet often overlooked, property. They demonstrated that many interpretability methods, including LIME, can produce drastically different explanations for very similar inputs, spurring research into metrics that explicitly measure an explainer’s resilience. Our work focuses specifically on empirically measuring the stability and robustness of our proposed method against these established baselines.

To ensure a consistent, interpretable, and universally applicable comparison across all evaluated methods (LIME, AKDE-LIME, TreeSHAP, and Anchor), we selected metrics tailored to their respective output formats, prioritizing direct measurement of change magnitude. Lower values indicate better performance (higher stability/robustness) for both chosen metrics:

**For Feature Attribution Methods (LIME, AKDE-LIME, SHAP)**: We measure the Mean Absolute Difference (MAD) between the feature importance weights of the base explanation (w) and the noisy explanation ($w{\;}^{\prime }$), Equation 9.

(9) $$D_{\mathrm{MAD}}(w,w{\;}^{\prime })=\frac{1}{M}\sum_{i=1}^{M}|w_{i}-w{{\;}^{\prime }}_{i}|$$

where M is the total number of features. This metric provides a direct measure of the average change in feature importance values.

**For the Rule-Based Method (Anchor)**: We measure the dissimilarity between the base rule set (A) and the noisy rule set (B) using the Jaccard Distance, Equation 10.

(10) $$D_{J}(A,B)=1-\frac{|A\cap B|}{|A\cup B|}$$

This metric quantifies the overlap between the two sets of rules, where a score of 0 indicates identical rules and 1 indicates no common rules.

This protocol allows for a direct and fair comparison of how much each explanation method’s output deviates when faced with small, controlled changes to the input data.

### Comparative overview of XAI methods

To ensure a fair and comprehensive comparison, we established a rigorous experimental setup. Before detailing the hyperparameter tuning and evaluation protocol, we summarize the key characteristics and comparative features of the XAI methods under investigation (LIME, TreeSHAP, Anchor, and our proposed AKDE-LIME) in Tables 1, 2 , and 3. This provides context for the subsequent quantitative evaluation of their stability and robustness.

**Table 1.** High-level comparison of key XAI properties. A check mark (✓) indicates native support; a cross (✗) indicates limited or no support.

Method	Fidelity ($R^{2}$) (Tulio Ribeiro, Singh, and Guestrin 2016)	Stability (Zhou, Hooker, and Wang 2021)	Robustness (Alvarez-Melis and Jaakkola 2018)	Precision & Coverage (Tulio Ribeiro, Singh, and Guestrin 2018)	Actionability (Wachter, Mittelstadt, and Russell 2017)	Reference
LIME	✓	✓	✓	✗	✗	(Tulio Ribeiro, Singh, and Guestrin 2016)
AKDE-LIME	✓	✓	✓	✗	✗	This work
TreeSHAP	✓	✓	✓	✗	✗	(Lundberg and Lee 2017)
Anchor	✗	✓	✓	✓	✗	(Tulio Ribeiro, Singh, and Guestrin 2018)

**Table 2.** Comparative summary of XAI methods.

Method	What it foes	Key strengths	Key weaknesses	Reference
LIME	Approximates the black-box model locally with a simpler, interpretable linear function.	Provides intuitive and interpretable feature importance scores for any model type.	Can be sensitive to perturbations and the choice of kernel width, leading to unstable explanations.	(Tulio Ribeiro, Singh, and Guestrin 2016)
AKDE-LIME	Uses adaptive kernel density estimation to create a more representative local sample for weighting.	Designed to be more robust to noise and sampling randomness than standard LIME.	The resulting feature weights can sometimes be very small (e.g., 1e-06), which may require careful interpretation. Bigger latency that the other XAIs methods	This work
TreeSHAP	Measures the exact marginal contribution of each feature to a prediction based on Shapley values.	Theoretically grounded, providing consistent and globally interpretable feature importance values.	While locally accurate, the global nature of Shapley values may not always capture purely local decision boundaries.	(Lundberg and Lee 2017)
Anchor	Generates high-precision, rule-based IF-THEN explanations that are easy for users to understand.	Produces highly stable and precise rules that “anchor” a prediction locally.	Does not rank the importance of features; it only provides the conditions for the rule.	(Tulio Ribeiro, Singh, and Guestrin 2018)

**Table 3.** Feature-based comparison of evaluated XAI methods.

Factor	LIME	AKDE-LIME	TreeSHAP	Anchor
Handles Local Variability	X	✓(More stable)	X	✓ (Rule-based)
Feature Importance Stability	X	✓(Consistent)	✓(Global stability)	N/A
Adaptive Weighting of Data	X	✓(Uses KDE)	X	X
Handles Model Noise	X (Sensitive)	✓ (Kernel-based)	✓(Less sensitive)	✓
Interpretable	✓	✓	X	✓

#### A framework for evaluation

A comprehensive framework for evaluating explainers considers multiple desirable properties. *Fidelity* measures how accurately an explanation reflects the underlying model’s behavior (Guidotti et al. 2018). *Actionability* assesses whether an explanation provides clear steps a user can take, a property often associated with counterfactual explanations (Wachter, Mittelstadt, and Russell 2017). Other criteria include *precision* and *coverage*, which are central to rule-based explainers like Anchor (Tulio Ribeiro, Singh, and Guestrin 2018). Table 1 provides a high-level comparison of the methods evaluated in this paper across several of these key properties.

#### Strengths and weaknesses of the XAIs methods

By evaluating LIME, AKDE-LIME, TreeSHAP, and Anchor against these crucial metrics, our study contributes to the systematic assessment of XAI techniques, providing empirical evidence to guide practitioners in selecting the most reliable method for their needs.

To provide a concise overview of the baseline methods evaluated in this study and to position our proposed AKDE-LIME, Table 2 summarizes their operational principles, key strengths, and known weaknesses. This comparison highlights the trade-offs between different explanation paradigms and motivates the need for methods that specifically address the stability and robustness of local feature attributions.

#### Feature-based comparison of XAI methods

To further contextualize our contribution, Table 3 provides a direct feature-based comparison of the XAI paradigms investigated in this paper. This highlights the specific capabilities and limitations that motivate the development of more robust local explanation methods like AKDE-LIME.

## Experimental Setup

To ensure a fair and comprehensive comparison, we established a rigorous experimental setup. This section outlines the fine-tuning process for our proposed method’s hyperparameters and details the evaluation protocol used to measure the stability and robustness of all XAI techniques across the selected machine learning models.

### Illustrative use case scenario: explaining energy load predictions

To contextualize the practical implications of different explanation properties, particularly stability, let us consider a specific scenario within the steel industry context. Suppose an energy management engineer uses one of the trained tree-based models (e.g., RandomForest) to predict the energy load type (“Light,” “Medium,” “Maximum”) based on current operational parameters.

Consider an instance x representing specific operating conditions (e.g., high Usage_kWh, late NSM – seconds past midnight) for which the model confidently predicts “Maximum Load.” The engineer wants to understand *why* the model made this prediction, perhaps to identify key drivers for optimization or anomaly detection. They employ the different XAI tools:

- **LIME**: Running LIME might produce an explanation like: “*Usage_kWh (+0.6), NSM (+0.3), and Lagging_Current_Power_Factor (−0.1) were the top factors pushing the prediction towards ‘Maximum Load’*.” However, due to LIME’s known sensitivity to perturbation sampling, running it again on the *exact same instance* x might yield slightly different weights or even a different ranking, perhaps showing “*NSM (+0.55), Usage_kWh (+0.4), …* .” This inconsistency is precisely the information drawback LIME suffers from – the engineer receives conflicting signals about the primary drivers for the same event, potentially undermining trust and making it difficult to decide which factor to prioritize for intervention.
- **TreeSHAP**: Applying TreeSHAP would provide stable feature contributions, perhaps “*Usage_kWh (+0.55), NSM (+0.28), …* .” These values are consistent and reflect the feature’s average marginal contribution across potential feature orderings. While robust and valuable, SHAP values don’t always capture purely local, possibly non-linear interactions in the same way a local surrogate model aims to, and their exact game-theoretic interpretation can be less intuitive for some users than a direct local linear approximation.
- **Anchor**: Anchor might return a rule like: “*IF Usage_kWh > 100 kWh AND NSM > 70000 seconds THEN predict ‘Maximum Load’ with 96% confidence*.” This provides a clear, highly stable condition sufficient for the prediction. However, it doesn’t offer a complete picture. What if Usage_kWh was only slightly lower? How important are other features like power factor outside of this specific rule? Anchor excels at providing sufficient conditions but lacks the ranked feature importance provided by attribution methods.
- **AKDE-LIME (Proposed)**: Our method, applied to the same instance x, would also produce feature importance scores, e.g., “*Usage_kWh (+0.58), NSM (+0.29), …* .” Crucially, due to the density-aware weighting, rerunning AKDE-LIME on instance x is expected to produce highly similar weights and rankings compared to the initial run, providing the engineer with a *consistent* local linear explanation.

**Criticality of Stability (User/Technical Perspective)**: The inconsistency demonstrated by standard LIME is critical. From a user perspective (the engineer), fluctuating explanations erode trust. If the perceived main driver changes between runs, the user cannot confidently rely on the explanation to take action (e.g., focus on reducing peak kWh usage vs. shifting operations based on time of day/NSM). Consistent explanations, as aimed for by AKDE-LIME and provided differently by TreeSHAP and Anchor, are essential for actionable insights.

From a technical perspective, stable explanations are crucial for model debugging and validation. If an explanation method gives unstable results, it’s hard to distinguish whether unexpected feature importance is due to a genuine model behavior that needs investigation or simply an artifact of the explanation method’s instability. AKDE-LIME aims to provide the intuitive local linear approximation of LIME but with enhanced reliability, making it more suitable for critical decision-making and model diagnostics. This illustrative scenario highlights the practical need for stable local explanations, motivating our focus on evaluating and improving this specific property.

### Hyperparameter tuning for AKDE-LIME

The performance of the AKDE-LIME method is influenced by several key hyperparameters that govern the perturbation process and the fitting of the local surrogate model. To determine the optimal configuration, we conducted an extensive grid search over the parameter space using the combined average of the Stability and Robustness scores as the primary optimization metric, where lower values indicate better performance.

The parameters tuned were:

- **KDE Bandwidth (bw)**: We explored both automatic rule-of-thumb methods (“scott,” “silverman”) and fixed scalar factors (0.5, 0.2) to assess the impact of the density estimation’s smoothing level.
- **Ridge Regularization (alpha)**: We tested different orders of magnitude (0.01, 0.1, 1) to find the optimal balance between the local surrogate model’s fidelity and its simplicity.
- **Perturbation Scale (perturb_std)**: We varied the scale of the neighborhood by testing factors of 0.01, 0.02, and 0.05 applied to the feature standard deviations.

The number of samples for perturbation was held constant at num_samples = 1000 and the distance clipping at the distance_clip_percentile = 99 to ensure a consistent basis for comparison.

Based on the results of this grid search, the optimal hyperparameter configuration that yielded the most stable and robust explanations (i.e., the lowest average deviation score) was found to be:

- num_samples: 1000
- bw: 0.2
- alpha: 1
- perturb_std: 0.01
- distance_clip_percentile: 99

All subsequent experiments reported in this paper utilize these optimized parameters for AKDE-LIME.

The combination of these optimal values provides insight into the behavior of our proposed method. The best performance was achieved by selecting the smallest tested values for the perturbation scale (perturb_std = 0.01) and the KDE bandwidth factor (bw = 0.2), along with the largest value for the Ridge regularization (alpha = 1). This suggests that the most stable explanations are derived by:

1. **Focusing on a very tight local neighborhood**: A small perturbation scale ensures that the surrogate model is trained only on samples immediately surrounding the instance to be explained, minimizing the influence of non-local model behavior.
2. **Emphasizing the most frequent distances**: A narrow KDE bandwidth creates a “peakier” density estimate, giving higher weight to perturbations that lie at the most common distances within this tight neighborhood.
3. **Promoting a simple surrogate model**: A high regularization penalty forces the linear model to be simpler, making it less likely to overfit to noise within the perturbed samples and their predictions, thus yielding more stable feature coefficients.

Together, these parameters create a highly localized and regularized explanation that is resilient to both sampling randomness and minor input perturbations.

**Figure 2.**
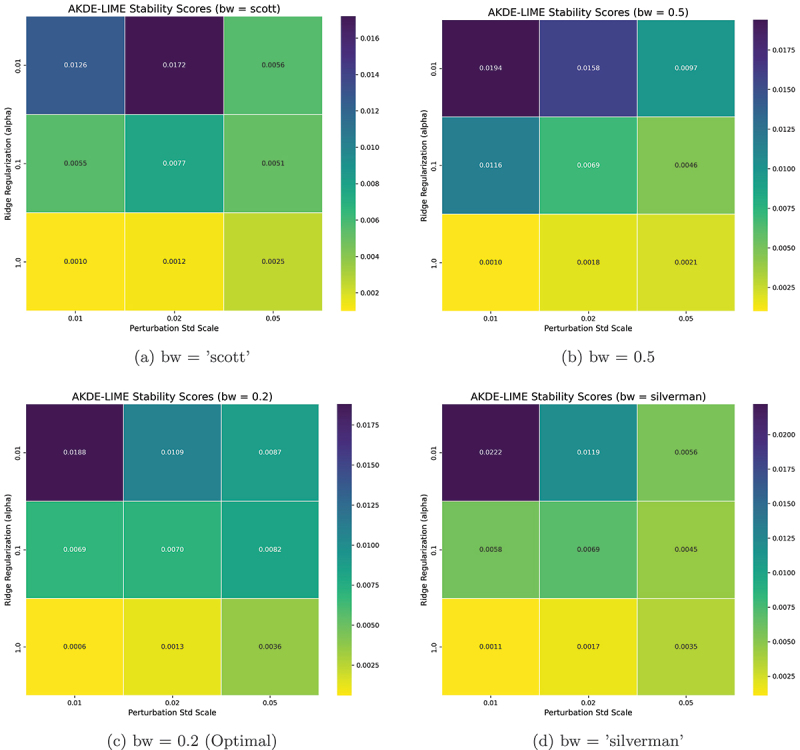
Heatmaps of AKDE-LIME stability scores across different hyperparameter settings. Lower scores (darker colors) indicate better performance. The optimal results were consistently found with a high alpha (1) and a low perturbation standard deviation (0.01), with the best overall score achieved using a bandwidth factor of 0.2.

**Figure 3.**
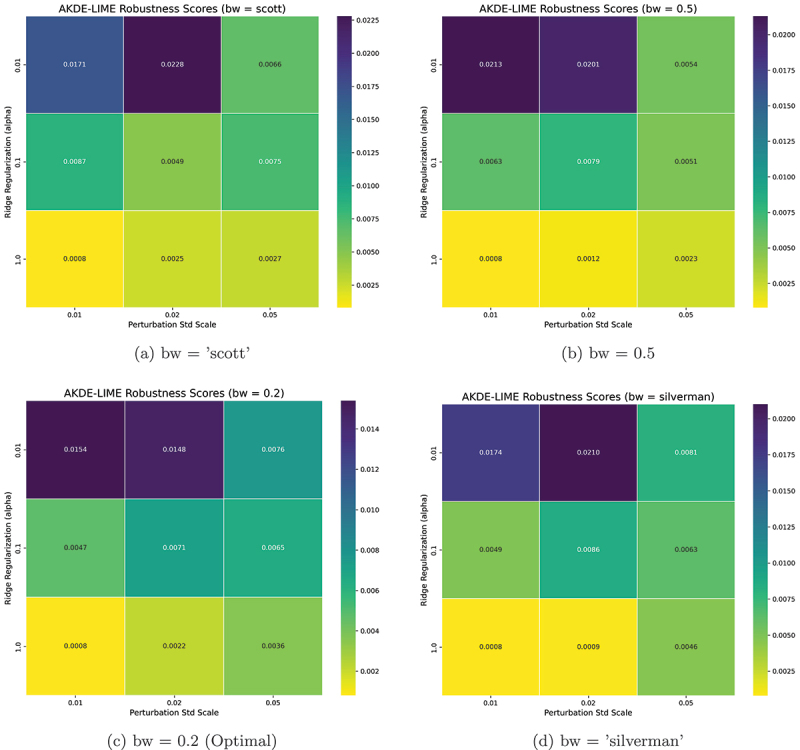
Heatmaps of AKDE-LIME robustness scores across different hyperparameter settings. The trend mirrors that of stability, with lower scores (darker colors) indicating better performance. The optimal configuration remains consistent, favoring high regularization and a highly localized neighborhood.

The performance landscape of our hyperparameter tuning for the AKDE-LIME method is visualized in Figures 2 and 3. These figures display heatmaps illustrating the sensitivity of the explanation quality to changes in the Ridge regularization penalty (α) and the perturbation scale (perturb_std) across the four different bandwidth (bw) methods tested. In these visualizations, lower scores (represented by darker colors) indicate better performance, corresponding to more stable and robust explanations.

The heatmaps clearly show a consistent trend: performance improves as the perturbation scale decreases and the regularization penalty increases. The optimal configuration, which corresponds to the cell with the lowest value (darkest color), was consistently found at perturb_std = 0.01 and alpha = 1. Among the bandwidth methods, bw = 0.2 yielded the overall best scores, leading to the selection of this configuration for all subsequent experiments.

### Performance evaluation results

#### Performance at 5% input noise level

**Figure 4.**
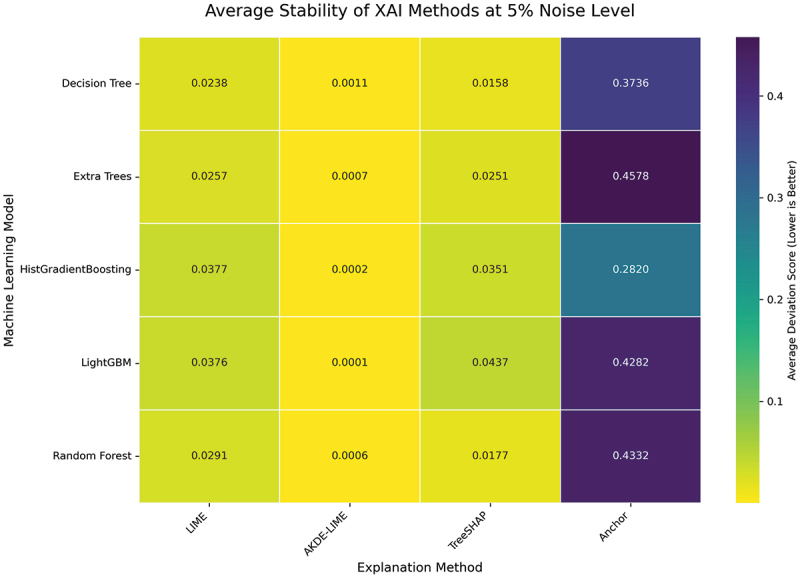
Average stability of XAI methods at 5% noise level. Lower scores (darker colors) indicate higher stability. AKDE-LIME consistently shows the lowest deviation scores across all models, outperforming standard LIME and often TreeSHAP.

**Figure 5.**
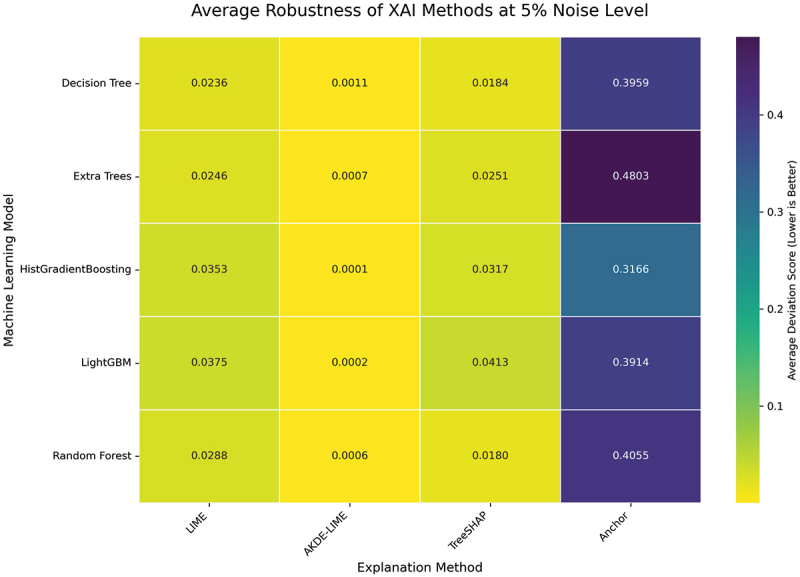
Average robustness of XAI methods at 5% noise level. The trend mirrors that of stability, with AKDE-LIME demonstrating superior robustness compared to LIME and competitive performance against TreeSHAP.

We begin by examining the performance of the explanation methods under a slight perturbation, where the noise level is set to 5% of each feature’s standard deviation. The results, averaged across all instance sizes (10 through 50), are visualized as heatmaps for both Stability and Robustness in Figures 4 and 5, respectively. In these figures, lower scores (represented by whiter colors) indicate better performance.

At this low level of input perturbation, several key trends emerge. Across all five machine learning models, our proposed AKDE-LIME method consistently and significantly outperforms standard LIME in both stability and robustness, demonstrating deviation scores that are an order of magnitude smaller. As visualized in Figure 4, the average stability scores for AKDE-LIME are exceptionally low (e.g., 0.0006 for Random Forest) compared to LIME (0.029).

Furthermore, AKDE-LIME achieves scores that are not only better than LIME but are often superior to those of TreeSHAP, a highly stable feature attribution method. For instance, when explaining the Decision Tree model, AKDE-LIME’s stability score is approximately 0.0011, whereas TreeSHAP’s is 0.0158. While Anchor, being a rule-based system, shows high stability (low Jaccard distance), its scores are not directly comparable to the feature attribution methods but are included for completeness. The results clearly indicate that the adaptive weighting mechanism in AKDE-LIME is highly effective at producing consistent and robust explanations, even when compared to state-of-the-art techniques.

#### Performance at 10% input noise level

**Figure 6.**
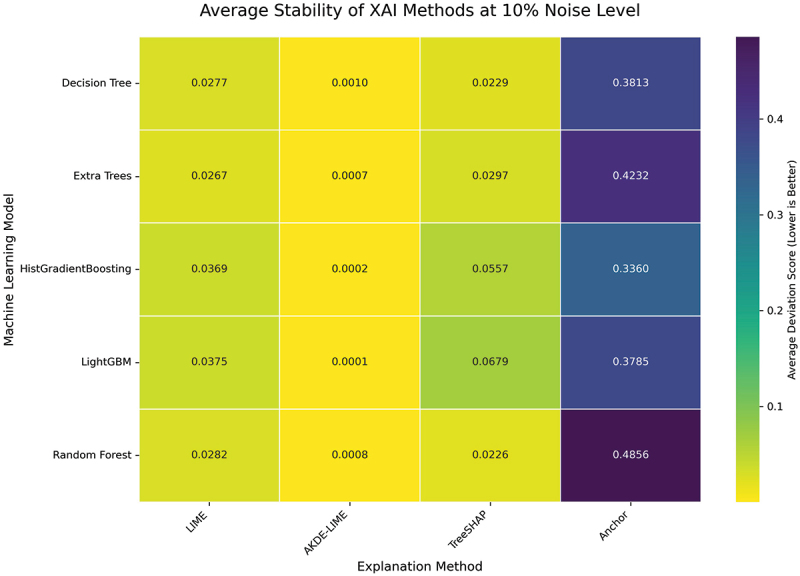
Average stability of XAI methods at 10% noise level. Even with increased noise, AKDE-LIME maintains exceptionally low deviation scores, indicating high stability.

**Figure 7.**
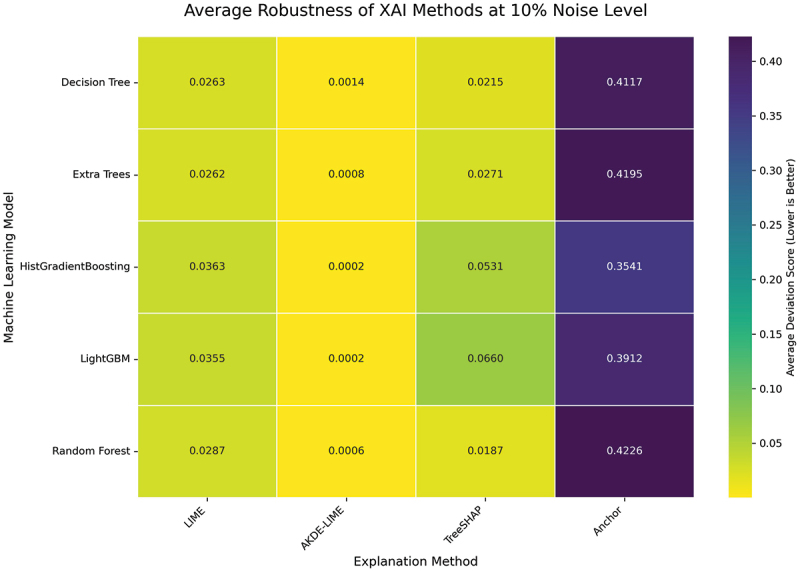
Average robustness of XAI methods at 10% noise level. AKDE-LIME continues to demonstrate the strongest robustness among the feature attribution methods.

Increasing the input perturbation to a 10% noise level allows us to assess the resilience of each explanation method under more significant data variation. The average Stability and Robustness scores for this experimental condition are presented in the heatmaps in Figures 6 and 7.

As shown in Figures 6 and 7, the performance trends observed at the 5% noise level largely persist, even with the increased input noise. Our proposed AKDE-LIME method continues to exhibit superior stability and robustness, consistently outperforming standard LIME by a significant margin across all five machine learning models. For instance, when explaining the Random Forest model, the average robustness score for AKDE-LIME is approximately 0.0006, whereas LIME’s score is 0.0288—a difference of nearly 50-fold.

Compared to TreeSHAP, AKDE-LIME also maintains a competitive edge, generally producing lower (better) deviation scores in both metrics. For example, for the LightGBM model, AKDE-LIME’s stability score is 0.0001, while TreeSHAP’s is 0.0680. This suggests that the density-aware weighting scheme is not only beneficial compared to LIME’s proximity kernel but also effective at producing highly consistent explanations that rival or exceed the stability of state-of-the-art, model-specific methods. The results at this noise level further validate the hypothesis that incorporating local data density into the weighting process leads to more reliable local explanations.

#### Performance at 15% input noise level

**Figure 8.**
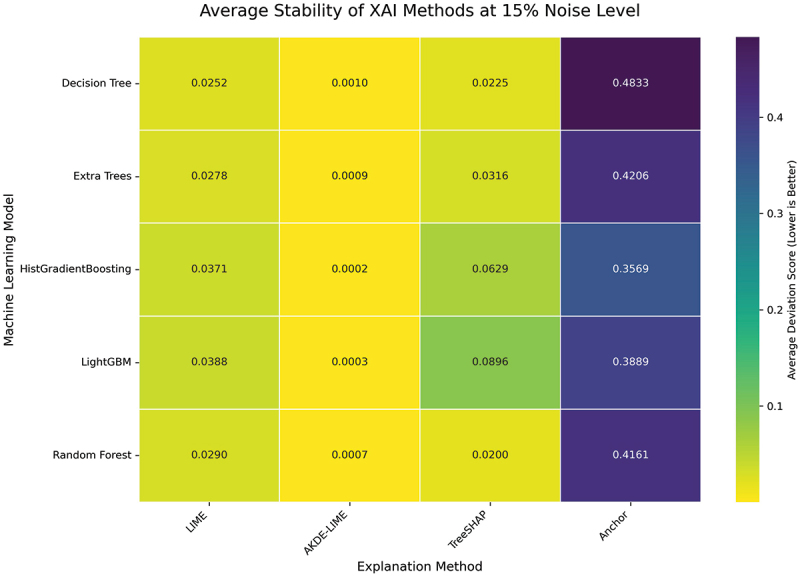
Average stability of XAI methods at 15% noise level. AKDE-LIME continues to exhibit the highest stability, with scores remaining exceptionally low despite the increased noise.

**Figure 9.**
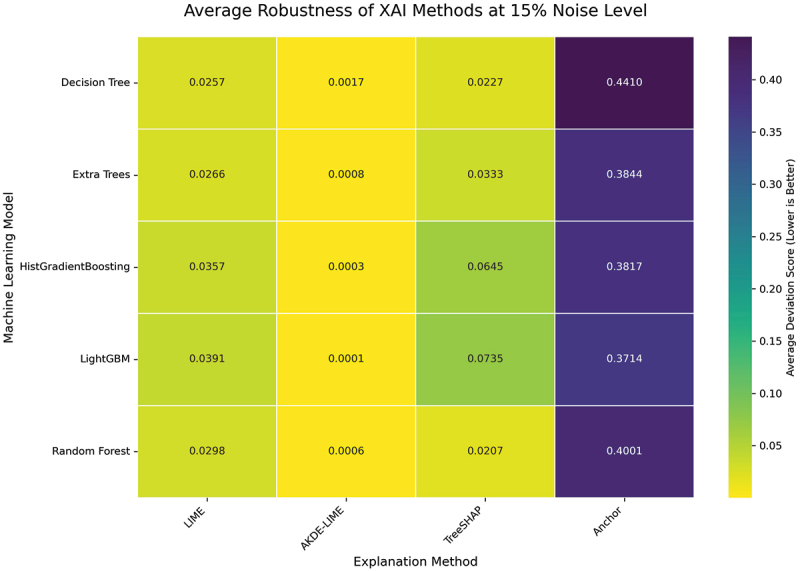
Average robustness of XAI methods at 15% noise level. The robustness of AKDE-LIME remains superior, showing minimal degradation compared to other feature attribution methods.

To further test the limits of each explainer’s resilience, we increased the input perturbation to a 15% noise level. This condition challenges the methods to maintain consistent explanations in the presence of more substantial data variance. The averaged results for this experiment are visualized in the heatmaps presented in Figures 8 and 9.

The results at a 15% noise level reinforce the trends observed previously. As shown in Figure 8, our proposed **AKDE-LIME** method maintains its superior performance, with stability scores that are consistently an order of magnitude lower than those of standard LIME and generally lower than TreeSHAP across all tested machine learning models. For example, when explaining the Extra Trees model, AKDE-LIME’s average stability score is approximately 0.0009, compared to 0.0278 for LIME and 0.0316 for TreeSHAP.

Similarly, the robustness heatmap in Figure 9 confirms the resilience of our method. While the deviation scores for LIME and TreeSHAP show a noticeable increase with the higher noise level, AKDE-LIME’s scores remain remarkably low (e.g., 0.0002 for HistGradientBoosting). This indicates that the adaptive density-based weighting is not just a minor improvement but a fundamental enhancement that effectively insulates the explanation from significant input noise. The consistent, state-of-the-art performance of AKDE-LIME at this noise level further strengthens the conclusion that it is a highly reliable method for generating trustworthy local explanations.

#### Performance at 20% input noise level

**Figure 10.**
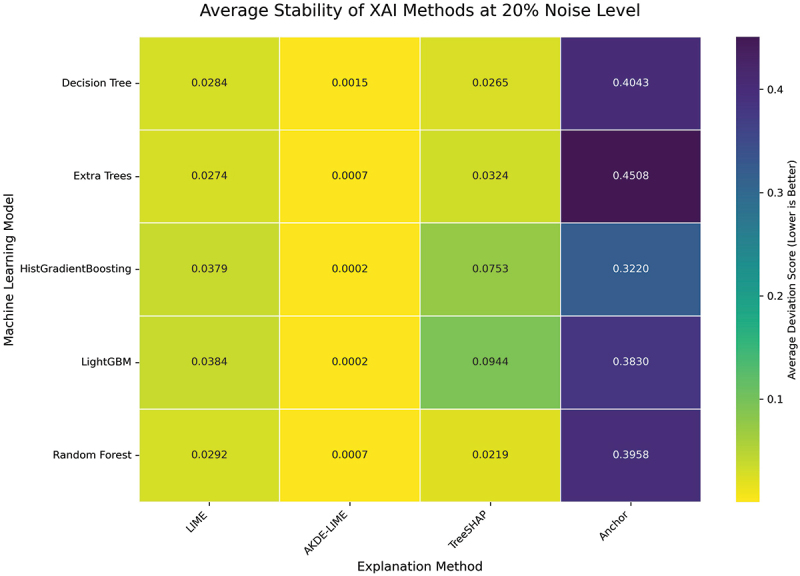
Average stability of XAI methods at 20% noise level. At the highest noise level, AKDE-LIME’s stability remains exceptionally high, showcasing its resilience.

**Figure 11.**
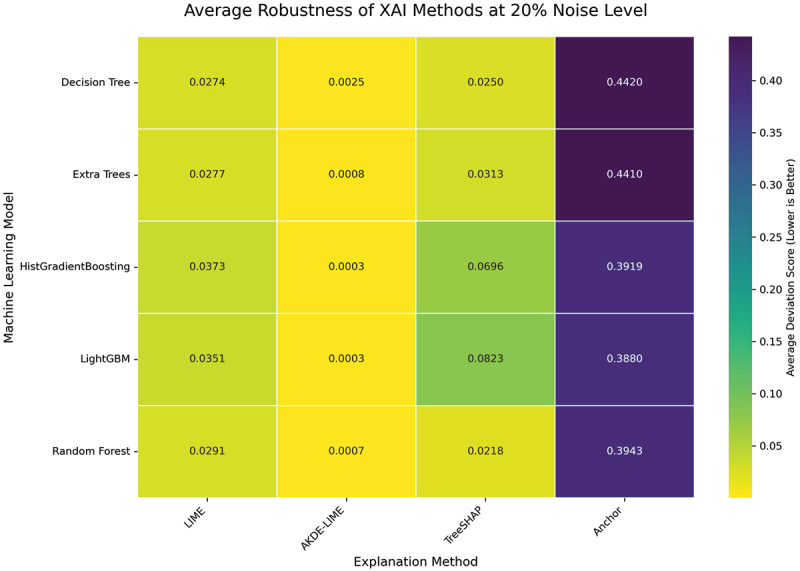
Average robustness of XAI methods at 20% noise level. The robustness of AKDE-LIME shows minimal degradation, continuing to outperform other feature attribution methods significantly.

The final set of experiments subjects the explanation methods to the highest level of perturbation in our study, with a 20% noise level. This condition serves as a stress test to evaluate the upper bounds of each method’s reliability. The averaged Stability and Robustness scores are summarized in the heatmaps in Figures 10 and 11.

Even at a substantial 20% noise level, the performance hierarchy established in the previous experiments remains firmly in place. The heatmaps in Figures 10 and 11 demonstrate the remarkable resilience of our proposed **AKDE-LIME** method. While the deviation scores for LIME and TreeSHAP continue to degrade with the increased noise, AKDE-LIME’s scores remain exceptionally low, often close to zero. For instance, when explaining the HistGradientBoosting model, AKDE-LIME’s average stability score is approximately 0.0002, compared to 0.0380 for LIME and 0.0755 for TreeSHAP.

This consistent, state-of-the-art performance under significant input perturbation provides strong evidence that the density-aware weighting scheme is the key factor in stabilizing the local surrogate modeling process. By focusing on a tight, representative neighborhood and promoting a simple linear explanation, AKDE-LIME effectively filters out the impact of input noise, leading to highly reliable and trustworthy explanations. This makes it a particularly suitable method for applications where model explanations must remain consistent even when dealing with noisy or slightly varied input data.

### Summary of experimental findings

Across all tested machine learning models and noise levels, our experiments reveal a clear and consistent performance hierarchy among the evaluated XAI methods. The key findings from our stability and robustness evaluations are summarized below:

- **Superior Performance of AKDE-LIME**: Our proposed **AKDE-LIME** method consistently demonstrated the highest levels of stability and robustness. In nearly every experiment, it achieved the lowest deviation scores, often by an order of magnitude compared to standard LIME (as seen in Figures 4–11). This indicates that its density-aware weighting scheme is highly effective at mitigating the impact of both sampling randomness and input perturbations.
- **Instability of Standard LIME**: As hypothesized, standard LIME produced the least stable and robust explanations among the feature attribution methods. Its performance degraded relative to other methods as the input noise level increased, highlighting its sensitivity to perturbations.
- **Strong Performance of TreeSHAP**: As a state-of-the-art, model-specific method, TreeSHAP exhibited strong stability and robustness. However, **AKDE-LIME** was frequently competitive with and, in many cases, superior to TreeSHAP, particularly when explaining the more complex gradient boosting models like LightGBM and HistGradientBoosting.
- **High Stability of Anchor**: The rule-based Anchor method also produced highly stable explanations, often yielding very low deviation scores. This is expected, as its rule-based output is inherently less granular than feature importance scores. However, its primary limitation remains that it does not provide feature attributions, making it suitable for a different type of explanatory goal.

In conclusion, the empirical results strongly support our central hypothesis. By incorporating a kernel density estimation into its weighting mechanism, **AKDE-LIME** successfully addresses the well-documented instability of LIME, providing a significantly more reliable method for generating local model explanations without sacrificing its model-agnostic flexibility.

## Limitations

While our study provides strong evidence for the enhanced stability and robustness of **AKDE-LIME**, we acknowledge several limitations that define the scope of our findings and offer avenues for future research.

- **Domain and Dataset Specificity**: Our empirical evaluation was conducted on a single, real-world tabular dataset from the steel industry. Although this provides a practical and relevant use case, the performance and relative ranking of the XAI methods may vary across different domains (e.g., finance, healthcare) or data modalities (e.g., text, images). Future work should validate these findings on a broader range of datasets to assess the generalizability of our conclusions.
- **Scope of Machine Learning Models**: The scope of our black-box models was intentionally focused on tree-based ensembles, which are prevalent in many practical applications. However, this study does not evaluate the performance of the explainers on other important classes of models, such as deep neural networks (DNNs), support vector machines (SVMs), or other non-tree-based architectures. The interaction between AKDE-LIME and these models remains an open area for investigation.
- **Focus on Specific Evaluation Metrics**: Our evaluation framework concentrates on the critical properties of stability and robustness, which are essential for user trust. However, other important dimensions of explanation quality, such as fidelity (how accurately the local surrogate represents the black-box model) and actionability (how useful the explanation is for guiding user decisions), were not empirically measured in this study. A more holistic comparison would benefit from incorporating a wider suite of evaluation metrics.
- **Hyperparameter Sensitivity**: Our study utilized the optimal hyperparameters for AKDE-LIME determined through a dedicated fine-tuning process, while standard parameters were used for baseline methods. A more exhaustive study could explore the sensitivity of all evaluated explainers to their respective hyperparameters (e.g., kernel width for LIME, threshold for Anchor), as this could influence their relative performance under different configurations.

These limitations notwithstanding, our work establishes a robust foundation for the utility of density-aware weighting in local explanations and provides a clear direction for extending this research to new models, domains, and evaluation criteria.

## Conclusions

In this paper, we introduced AKDE-LIME, a novel local explanation method designed to address the well-documented instability of the standard LIME algorithm. By incorporating a density-aware weighting scheme that combines Kernel Density Estimation with a proximity-based kernel, our method produces more stable and robust local explanations. We conducted a comprehensive set of experiments to validate our approach, comparing AKDE-LIME against LIME, TreeSHAP, and Anchor across five different tree-based models under varying levels of input noise.

Our empirical results lead to several key conclusions. First, AKDE-LIME consistently and significantly outperforms standard LIME in both stability and robustness across all tested models and noise conditions. The deviation scores for our method were often an order of magnitude lower, confirming that the adaptive weighting mechanism successfully mitigates the impact of sampling randomness and input perturbations. Second, the performance of AKDE-LIME was frequently competitive with, and in many cases superior to, TreeSHAP, a highly efficient, model-specific explanation method. This demonstrates that our model-agnostic approach can achieve state-of-the-art stability without sacrificing its broad applicability. Finally, our hyperparameter tuning revealed that the most reliable explanations were generated by focusing on a highly localized neighborhood and using a simple, regularized surrogate model, validating the core hypothesis of our method.

The findings of this study suggest that AKDE-LIME is a more reliable and trustworthy alternative for generating local explanations, particularly in practical scenarios where data may be noisy or where consistency is critical for user trust.

Future work should aim to extend the validation of AKDE-LIME to other domains and data modalities, such as text and image data. Furthermore, evaluating its performance on other classes of machine learning models, including deep neural networks, would be a valuable next step. Finally, expanding the evaluation framework to include metrics for other desirable properties, such as fidelity and actionability, would provide an even more holistic understanding of the method’s overall quality and utility.

## Data Availability

The data that support the findings of this study are openly available in the UCI Machine Learning Repository at https://doi.org/10.24432/C52G8C., reference number (V E, Shin, and Cho 2021).
